# Digital open science—Teaching digital tools for reproducible and transparent research

**DOI:** 10.1371/journal.pbio.2006022

**Published:** 2018-07-26

**Authors:** Ulf Toelch, Dirk Ostwald

**Affiliations:** 1 QUEST Center for Transforming Biomedical Research, Berlin Institute of Health, Berlin, Germany; 2 Biological Psychology und Cognitive Neuroscience, Freie Universität Berlin, Berlin, Germany; 3 Computational Cognitive Neuroscience, Department of Education and Psychology, Freie Universität Berlin, Berlin, Germany; 4 Center for Cognitive Neuroscience Berlin, Freie Universität Berlin, Berlin, Germany; 5 Center for Adaptive Rationality, Max Planck Institute for Human Development, Berlin, Germany

## Abstract

An important hallmark of science is the transparency and reproducibility of scientific results. Over the last few years, internet-based technologies have emerged that allow for a representation of the scientific process that goes far beyond traditional methods and analysis descriptions. Using these often freely available tools requires a suite of skills that is not necessarily part of a curriculum in the life sciences. However, funders, journals, and policy makers increasingly require researchers to ensure complete reproducibility of their methods and analyses. To close this gap, we designed an introductory course that guides students towards a reproducible science workflow. Here, we outline the course content and possible extensions, report encountered challenges, and discuss how to integrate such a course in existing curricula.

## The need for open science education

The scientific process from idea to publication is complex and seldomly reflected in full in traditional research articles [1]. As of 2015, only 13% of research articles include raw data [2], and even fewer include data analysis code [3]. The reasons for this are numerous, such as unclear data protection issues, pending patents, or lack of technical know-how [4]. Transparency of this process is, however, desirable, as an in-depth evaluation of presented evidence depends on a detailed account of methods and results. A lack of such an account potentially results in difficulties in replicating results or impairs future research that builds on limited evidence. This is particularly prevalent in biomedical research, in which clinical findings rest on a chain of previous results from in vitro and in vivo experiments [5,6]. Incomplete reporting can result in unnecessary costs and, at its worst, endanger patients’ lives [7]. As a response to these problems, journals [8], funding agencies [9,10], and policy makers have introduced more or less strict regulations for how hypotheses, materials, data, and procedures should be made available. These new requirements, which come under the umbrella term ‘open science’, have a direct impact, particularly on the work of young researchers: they must ensure that their research practices are in line with these new requirements to be able to publish their work or apply for funding. At the same time, open science practices can also directly benefit young researchers by increasing their visibility and expanding their network. There is some evidence that articles with open access gain more citations, particularly when associated data is also published openly [11]. Moreover, researchers may also benefit from the availability of scientific data and code in their own research projects.

As a result of these developments and the changing scientific landscape, new and established digital tools have evolved into a novel toolbox for digital open science. In our view, however, the diffusion of these innovations is somewhat hindered, as they are seldom part of a curriculum in the life sciences. An EU report recently declared early career education in open science highly desirable, but ‘training opportunities for open access and open data are not yet widely offered’ [11]. There are notable initiatives that offer educational programs regarding open science practices [12,13]. Whereas these initiatives offer dedicated workshops that are often given off site, we designed a university-level course that can be adapted to existing curricula early on, i.e., at the MSc and early PhD level. An integral and distinguishing part of our course is the direct practice of acquired skills. This is achieved by employing these skills in local research projects with resident researchers. Hereby, students get into contact with everyday problems of scientists such as messy data and unannotated Excel sheets. They experience directly how to meet the quality standards of a reproducible and transparent research process [14]. Course participants were expected to have only minimal programming experience, and the course was designed without restrictions to any particular programming language. Whereas this course was mainly designed for students in the life sciences, some students from the humanities took part as well. In the following, we outline the curriculum of the course, which comprises approximately 60 hours with 15–20 hours of lectures and tutorials. Additionally, we will highlight tools and techniques that are supplementary and could serve as material for more advanced classes. All materials for this course, including a reading list and presentations, are available as a public project on the Open Science Framework (OSF) (https://osf.io/X6892/).

## Course overview

**Fig 1:**
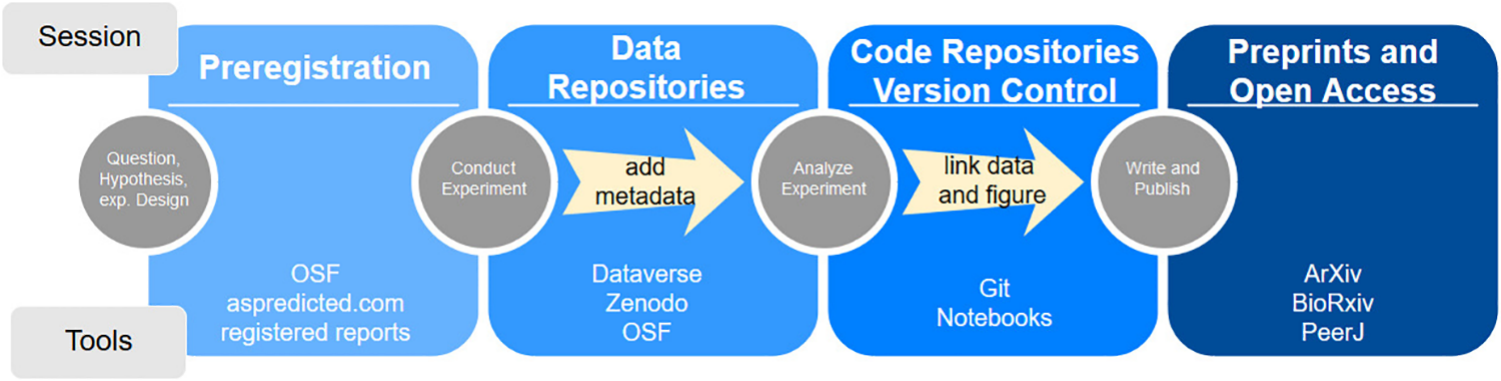
Outline of interactive teaching sessions and associated tools. OSF, Open Science Framework.

As mentioned above, our course consisted of two parts. The first part conveyed the theoretical background of open science and comprised an introduction to the available digital tools in interactive teaching sessions (Fig 1). To introduce students to the open science toolbox, we developed a narrative for this part of the course that involved a planned replication of an existing study. Students needed to identify and study a paper from their field of expertise or interest with a single, ideally simple, experiment that should be replicated. All content was then practiced on this paper. For example, when participants simulated data, they simulated data for the experiment in the paper they selected for the course. In the spirit of open science, tools in the course were selected for their free accessibility and were ideally open source and noncommercial. In some instances, such as the commercial GitHub platform, we had to compromise: over the last years, GitHub has established itself as the de facto community standard for online version control with Git. We also mentioned alternatives, such as an open source GitLab community edition on a self-hosted server. However, such open-source alternatives often require considerable expertise and dedication to establish and maintain at an academic institution. In future instantiations of our course, we will include some guidance on how to select open science tools from both an idealistic and a pragmatic perspective. The second part involved projects in which students applied digital open science tools to a research project of their choice. This resulted in a symposium at which students presented their projects.

## Interactive teaching sessions

In the following, we will briefly outline each session of the first part of the course. A detailed description of the background and exercises are available in the associated OSF project. For brief explanations of some of the open science key terms, see Table 1.

**Table 1:** Definitions of some key terms in open science practice and education.

Preregistration	The practice of digitally registering an in-depth data analysis plan before data acquisition. Preregistration allows for clearly separating confirmatory hypothesis-testing from exploratory hypothesis-generating research [31,32].
Registered Report	Registered reports are research papers whose potential for publication is evaluated by means of peer review and editorial decisions prior to data collection. As such, these reports emphasise the importance of the research question and methodological quality independent of the actual results [15].
*p*-hacking	The use of data mining techniques to uncover patterns in data that are “statistically significant” based on the use of *p*-values, but for which no pre-existing causal hypotheses were devised [16].
HARKing	The practice of Hypothesising After Results are Known in scientific writing [17].
Green vs. Gold OA	Green OA refers to the practice of making a copy of a published journal article openly available via an online repository or personal website. Gold OA refers to the availability of journal articles from the journal’s website, sometimes based on additional article processing charges required by the publisher.

**Abbreviations**: HARKing, Hypothesising After Results are Known; OA, open access.

### Introduction

In the first session, we recapitulated the steps involved in a scientific project, including an introduction to the OSF [14] and an outline of the expected active contributions of the students.

### Preregistration

Preregistration is a summary of the research rationale, hypotheses with predictions, methods, and, in an extended version, also an analysis plan [15]. We introduced students to the minimum requirements for a preregistration and the difference between preregistrations that are either submitted directly to a journal (registered reports) and submissions to a suitable platform like OSF or aspredicted.com. We discussed how preregistration potentially prevents *p*-hacking [16] and Hypothesising After Results are Known (HARKing) [17], two major threats for the reproducibility of science [18]. Furthermore, we emphasised the difference between confirmatory and exploratory analysis and particularly why exploratory analysis is not per se problematic but can be a major driver of scientific discovery [19].

### Data repositories

Once data has been collected, it has to be saved in a digital format in an appropriate repository. We highlighted different types of general repositories (OSF, Dataverse, Zenodo) versus specialised repositories for a particular data structure (for example, proteins [20] or MRI data [21]). Importantly, all repositories should ideally adhere to the ‘FAIR’ guiding principles: Findability, Accessibility, Interoperability, and Reusability [22]. This includes unique digital object identifiers, but also metadata that allows humans and machines to understand the data format. Additionally, we also covered differences between licences for the shared data and legal and ethical issues that are often associated with data from human subjects.

### Code repositories, version control, and notebooks

Raw data are seldom reported in a paper but rather are derived statistics and figures of aggregated data. Annotated analysis scripts make the transition from raw data to figure transparent and can be shared via specialised code repositories. One major advantage of such repositories is a version control system that keeps track of changes to the code and allows for collaborative work on the scripts. In particular, we introduce students to Git [23] as a software version control system that connects to online repositories like GitHub and Gitlab [24–26]. We then integrated analysis code with annotating text into notebooks (livescript for MATLAB, Jupyter for Python, and RMarkdown for R) to illustrate how to write a fully reproducible analysis in a manuscript format.

### Open access

The final step in the scientific process is the publication of the results (and, of course, the data and data analysis steps that led to the results) in a journal. In this session, we gave an introduction to the publication process and explained differences in open access formats. We highlighted different monetisation schemes of publishers (for example, pay for view versus pay for publication). Moreover, we discussed university- or funding agency-specific regulations on who is paying for open access and included some cautionary notes on predatory open access journals [27]. Finally, we covered the differences between preprint servers, green and gold access, and how they fit into the current publication landscape [28–30]. This session should enable young researchers to discuss with their supervisors which journals may be a suitable outlet for their work under consideration of publications costs. A possible extension, not yet covered in our course, may include public copyright licencing schemes for research materials, such as the Creative Commons licence suite (https://creativecommons.org/).

### Chances and limitations of open science

The last session was dedicated to the promises and limitations of open science and directly linked to the discussion on open access publishing. Students collected advantages of open science and arguments under which circumstances there are limitations to open science in small groups and presented them. Important topics included privacy concerns for patient and participant data and patents. Students further listed the fear of being scooped before their own publication and a reuse of data they collected without proper attribution. Finally, we discussed arguments for convincing a potential PhD supervisor to embrace open science principles.

## Project work

In a final session, students presented their plans for an open science project that they would conduct over the next couple of weeks. Importantly, these projects were directly connected with current or past research projects in our department. The projects were roughly separable into two categories: students either came up with an idea for a small replication experiment that they conducted and analysed, while others worked on an already-collected data set. Importantly, students actively used a selection of the aforementioned open science tools in these projects. Example projects include data set projects in which students transformed already existing neuroimaging data sets into a novel community standard for data sharing (the Brain Imaging Data Structure (BIDS, [33], see below). Another project involved an extended replication of an experiment and making resulting data for this available on OSF (https://osf.io/jaxdp). Yet another project extended an analysis of an existing data set from a bachelor thesis and used Git as a version control system for programming an analysis on participants’ choice data. All these projects enabled students to use open science tools in a scientific context and integrate open science practices in their workflows. This setup is, in our opinion, feasible in almost every environment, as many labs have unpublished data sets and undocumented analyses or are interested in a small replication study. This directly connects teaching to the scientific process, enabling students to contribute to knowledge creation in a meaningful way and hence increasing motivation [34–37]. We concluded the course with a colloquium at which students gave short presentations on their projects and reflected on challenges they encountered during the project. In the following, we discuss a concrete example project in further depth.

### An example project

In the past, many cognitive neuroimaging labs, including our own, have published the results of neuroimaging without concurrently making the raw data and analyses code readily available for other researchers. While data sharing may still have happened in an informal manner by request to the author and in the idiosyncratic format that the data set was saved in, two recent developments have made ‘one-click’ sharing of neuroimaging data much more feasible. First, many general-purpose repositories, such as the OSF, have improved and now allow users to upload files of up to 5 GB, which covers the typical file sizes in neuroimaging. Secondly, the neuroimaging community has developed a common standard of how to organise functional neuroimaging data [33]. These two developments now ease the refurbishment of old data sets according to the open science standard. In a concrete example, Ostwald and colleagues [38] recorded a large EEG and simultaneous EEG–fMRI data set of human participants performing a simple visual perceptual decision task. In the original paper, the data were analysed from an information theoretic perspective and provided some evidence for the dynamic and distributed representation of decision-relevant information in the human brain. To make these data available for other researchers, Georgie and colleagues [39] converted the raw behavioural, EEG, and fMRI data recordings to the Brain Imaging Data Structure (BIDS) format. BIDS specifies in detail in which folder structure the data should be organised, how files should be named, which file types should be used, which information about the behavioural paradigms should be shared, and which metadata should be included. Converting an idiosyncratically organised data set into the BIDS standard is a programming exercise that is highly suited for MSc students developing their programming skills. In addition, basic analyses of the data set already reported in Ostwald and colleagues [38] were reproduced using newly developed code that maximises code readability. Finally, the data were uploaded as a project on the OSF, and a report describing the data set was submitted to a journal that specialises in data descriptors [40]. An important issue in sharing human biomedical data such as brain images is privacy protection. In general, it is unlikely that participants who took part in neuroimaging studies between 1995 and 2010 provided consent for the open sharing of their neuroimaging raw data, because this was a nonissue in obtaining study approval from local ethics committees. Honouring this fact, we decided to make the data available in a protected manner: fellow researchers who would like to assess and use the data for their own analyses are required to sign a data use agreement. With this agreement, data users, for example, commit to refrain from trying to identify individuals in the study or sharing the data further with third parties. In summary, refurbishing old data sets for the open science paradigm has many advantages in open science education: First, supervisors are familiar with the scientific matter at hand and can provide excellent support. Second, students carry out well-defined tasks with easily identified goals (conversion of a data set to a common standard, reproduction of previously performed data analyses). Third, students acquire an overview of the necessary steps to implement open science practices in novel projects and become aware of ethical and legal constraints such as data privacy concerns. Finally, students actively contribute to real and meaningful research projects and can obtain their first experiences in publishing scientific articles.

## Course evaluation

We evaluated the course in two steps. First, we asked for immediate feedback on teaching style and possible improvements. In a second step, we assessed how frequently course participants were able to integrate open science practices in their workflow. The first evaluation was conducted by the education quality assessment team at Freie Universität Berlin. It revealed that students particularly liked the project work (‘Encouraging students to do their own projects is an amazing idea’). Critical points involved the low number of sessions, which sometimes led to only superficial or too-rapid coverage of material. That is, pacing was not ideal for the diverse group of students. A further modularisation of content, particularly when it comes to version control and programming, may help ameliorate these problems. Beyond this, we felt that a course should address the immediate needs of participants. In our course, this was sometimes not feasible given that students from both the life sciences and the humanities took part. Based on this experience, we suggest that open science courses should be closely tailored to their audience. Our course is, against our initial intuition, not ideally suited for a blend of students from different disciplines, at least at this advanced level. In a slightly less elaborate form, it may be a good fit for a beginner’s course on good scientific practice.

**Fig 2:**
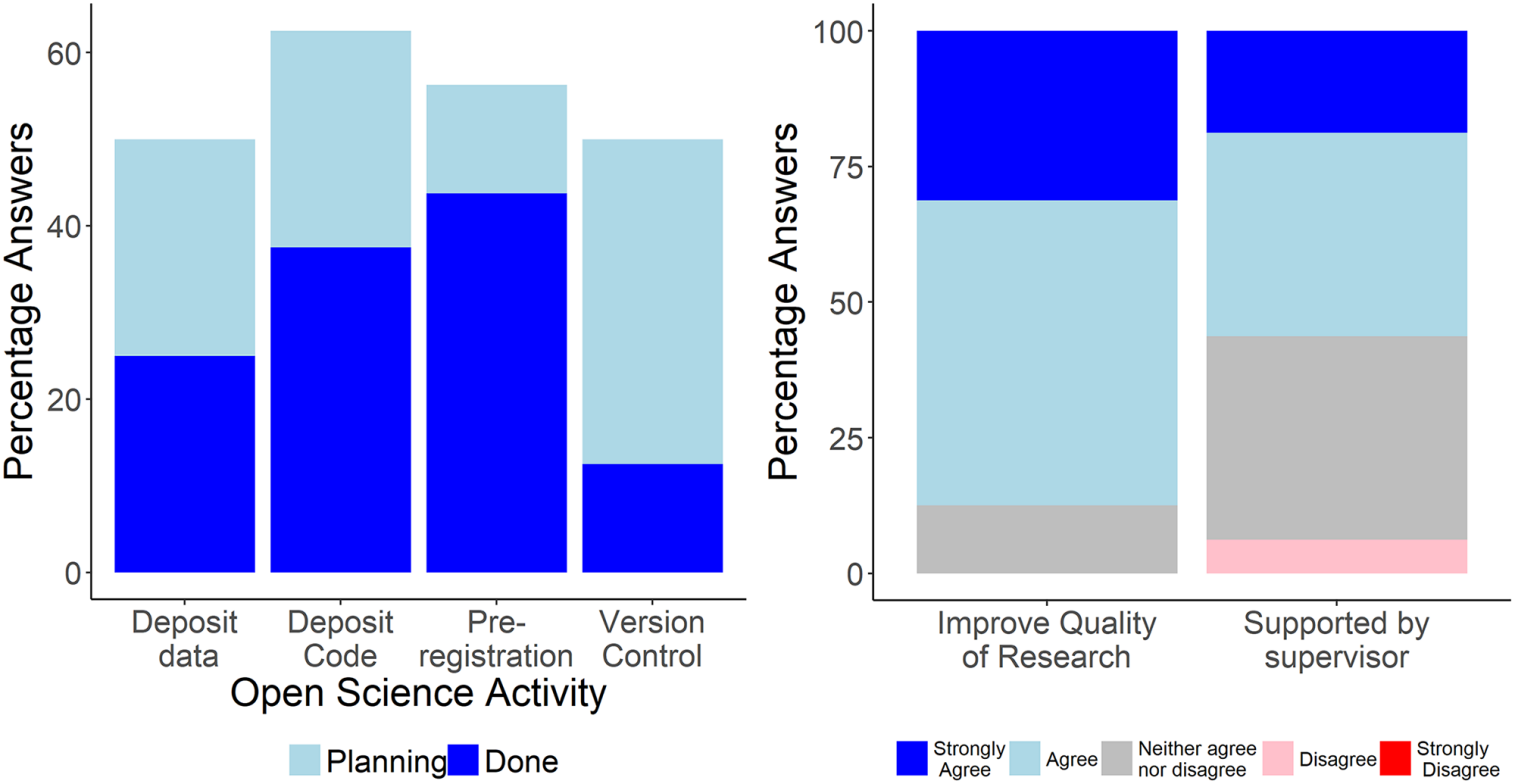
Post-course assessment of the impact on open science practices approximately half a year after the course. A. Percentage of students engaging or planning to in different open science practices. B. Student agreement on whether course content will improve the quality of their research and whether their supervisors support them in their open science endeavours. Both panels are based on a sample of N = 17 (https://doi.org/10.17605/OSF.IO/32T5H).

The best open science course is only an academic exercise if scientific behaviour after the course is not changed. To evaluate how our course affected student behaviour, we contacted students half a year after completion of the course. Out of 35 students enrolled in the course, 17 answered this survey (approximately 50%). Most students were psychology majors at the end of their MSc thesis or early in their PhD. We found that more than half of the responding students planned to or actually engaged in open science practices, with under 20% not planning to apply the learned skills (Fig 2A). An exception was version control: here, students were reluctant to use it, and 35% of them even had no plans to use it in the future. To adapt the course accordingly, we suggest three scenarios for teaching version control in future course iterations. First, version control teaching may be omitted from the course entirely. This could be particularly suitable for a shorter course in which it is unlikely that participants will engage in larger software projects. Second, only basic mechanisms of version control may be explained, and teaching will instead focus on writing reproducible code. With a focus on reproducible code, more time can be spent on important aspects such as coding conventions, commenting, and variable names, all of which foster the communicative aspects of code design. Finally, we think that in an advanced open science data analysis course, version control should be incorporated. Here, enough time should be allocated for learning techniques such as branching, merging, and pull requests.

Overall, however, above 80% of the responding students agree or strongly agree that the quality of their own research will be improved by open science practices (Fig 2B). Moreover, half of the students reported that their supervisors were supportive of open science (one student did not feel supported). These results suggest that a course on open science can raise awareness for open science as an important quality management tool. At the same time, continued support, infrastructures, and time are necessary to sustainably change behaviour and anchor these practices in daily practice. Of course, we should point out that we describe here a first instantiation of an open science course with a relatively small number of participants who mainly stem from one field (psychology). The evaluation data, even though promising, is thus only preliminary and not robust enough to warrant any policy implementations yet. It will take more data on future student generations to fully evaluate which methods will result in a sustainable implementation of open science practices in scientific workflows. Notably, the methods taught in this course aim at improving research quality, and with that ‘increase value and reduce waste’ [5]. Whether these goals will be met by teaching open science practices in the long run is beyond our current scope, but we believe that we should feel encouraged to design and evaluate educational programs with respect to these goals.

## Advice to others

With open science practices gaining more and more widespread acceptance, we presume that sooner rather than later, fellow academics will aim to establish open science teaching activities at their departments. We admit that this course was implemented in a favourable environment with high degrees of freedom for the lecturers, a supportive dean, and some intramural funding. Fortunately, supervisors of projects understood the importance of open science practices and gave students the opportunity to use data collected in their lab. Under less ideal conditions, we suggest starting small, with only one or two topics that can easily be integrated into the curriculum. To initiate this process, it may be helpful to identify faculty members who are already using or are at least sympathetic to open science methods and who may support students in open science projects. Implementation of an open science course will of course depend on departmental specificities, but in the following, we compiled some key points that may help others to integrate open science content into existing graduate curricula.

### Tie open science content to existing methods or soft-skill courses

We think that existing methods or soft-skill courses are highly suitable for the integration of open science education into existing curricula for MSc or PhD programs. For example, existing programming or data analysis courses can readily be adapted to incorporate aspects of the current open science landscape, such as the use of software version control system Git, and sharpen the students’ understanding that all scientific programming code is essentially part of the scientific communication process and hence needs to be written in a clear and understandable manner. Other targets for the incorporation of open science content can be existing soft-skill courses, for example on questions of research ethics. While traditional research ethics courses emphasise, for example, the pros and cons of animal experiments or the meaning of scientific fraud, these courses are equally suitable to discuss the ethical obligations involved in performing publicly funded research or the impact of preclinical reporting quality on patient welfare in clinical trials.

### Provide academic credit

We think that it is essential that open science content is tightly incorporated into existing curricula such that engaging with questions of open science is in direct partial fulfilment of the student’s degree. Open science content should not be regarded as an extracurricular activity, as this would undermine the students’ motivation and perception of open science as a novel working standard in academia. For example, in our case, we provided students with the opportunity to perform their open science projects in partial fulfilment of their neurocognitive methods class, which in the past had typically involved a programming exercise.

### Use scientifically meaningful, not play projects

As described in the example project above, we believe that open science education may also be able to contribute to improving the scientific literature in many fields. By enabling researchers to critically re-evaluate previously analysed data from an open science perspective, it helps students to become familiar with essential data analytical skills and potentially improve the scientific record.

### Exploit the rapidly expanding resources for open science education

The open science movement is rapidly expanding. Almost every week, new tools or resources that can help researchers to implement open science practices become available. Amongst these are also tools for open science education. For example, at present, both an open science training handbook [41] as well as open science online resources [42] are under development. We thus encourage potential open science educators to, for example, subscribe to open science email lists or follow open science experts on twitter, in order to remain updated about the availability of open science teaching tools.

## Concluding remarks

The course described here offers a first introduction to open science for early career researchers We hope that ideas and aspects of this course will find their way into higher education curricula. We acknowledge that starting new open science practices will initially increase the work and effort needed to complete a full research cycle from question to publication. This may put a burden on young researchers who already struggle with the other obstacles in their new career. It is in our view thus important that supervisors and PIs are supportive and allow for additional time to implement these practices. We want to stress that this will result in a win–win situation, because open science practices are an important part of quality management in science.

We have not established open science practices rigorously, if at all, in our own past research projects. Rigour and highest quality standards for knowledge creation, however, mandate a change in our thinking. This course, hopefully, provides some material to inspire teaching young researchers the necessary skills to implement open science practices and increase the sustainability and quality of modern science even beyond the prevailing high standards.

## Abbreviations

BIDS: Brain Imaging Data Structure
EEG: Electroencephalography
FAIR: Findability, Accessibility, Interoperability, and Reusability
fMRI: Functional Magnetic Resonance Imaging
HARKing: Hypothesizing After Results are Known
OSF: Open Science Framework
